# Supplementary material for: Supplementation with Vitis vinifera Jingzaojing Leaf and Shoot Extract Improves Exercise Endurance in Mice
Source: Nutrients. 2022 Sep 28;14(19):4033. doi: 10.3390/nu14194033 (PMC9573418; doi:10.3390/nu14194033)
Supplement: Supplementary file 1 [file nutrients-14-04033-s001.zip › nutrients-1932377-supplementary.pdf]

Supplementation with *Vitis vinifera* Jingzaojing leaf and shoot extract improves exercise endurance in mice

Contents

1. Supplementary Methods
2. Supplementary Table
3. Supplementary Figures

1. Supplementary Methods

Histology

Skeletal muscle tissues were immediately placed in 30% sucrose solution and embedded with liquid nitrogen-cooled isopentane. For succinate dehydrogenase (SDH) staining, frozen sections (10 μm) were incubated in 0.2 M sodium phosphate buffer solution (pH 7.6) containing 0.6 mM nitro blue tetrazolium and 50 mM sodium succinate (Sigma-Aldrich, St Louis, MO, USA) over 30 min at 37°C. Slides were washed with DiH_2O and mounted with aqueous mounting media. For staining of MHC isoforms, muscle sections were incubated overnight at 4°C with primary MyHC antibodies [MyHC I (#BA-D5), MyHC IIa (#SC-71) and MyHC IIb (#BF-F3), DSHB, lowa City, IA, USA]. After washing, secondary antibodies [Alexa Fluor 350-conjugated goat anti-mouse IgG2b (#A21140), Alexa Fluor 488-conjugated goat anti-mouse IgG1 (#A21121) and Alexa Fluor 594-conjugated goat anti-mouse IgM (#A21044), Thermo Fisher Scientific, Waltham, MA, USA] were incubated for 1 h at 37°C. Images were acquired using a Leica DM750 microscope (Leica, Wetzlar, Germany). Image analysis was performed using iSolution DT 36 software (Carl Zeiss, Oberkochen, Germany).

Western blotting

Tissue homogenates and cell lysates (20 μg) were separated by 10% Sodium dodecyl-sulfate polyacrylamide gel electrophoresis (SDS-PAGE) and transferred to Polyvinylidene fluoride or polyvinylidene difluoride (PVDF) membranes. After blocking with 5% skim milk, blots were probed with primary antibodies against Sirt6 (#12486, 1:200 dilution, Cell Signaling Technology, Beverly, MA, USA), CREB (#9197, 1:1,000 dilution, Cell Signaling Technology), p-CREB (#9198 1:1,000 dilution, Cell Signaling Technology), Sox6 (ab64946,

1:1,000 dilution, Abcam, Cambridge, UK), T-OXPHOS (ab110413, 1:1,000 dilution, Abcam), Mfn1 (ab57602, 1:1,000 dilution, Abcam), OPA1 (612606, 1:1,000 dilution, BD Biosciences, Franklin Lakes, NJ, USA), HSP90 (ADI-SPA-836-F, 1:1,000 dilution, Enzo Life Sciences, Plymouth Meeting, PA, USA), PGC-1α (AB-3242, 1:1,000 dilution, Millipore, Danvers, MA, USA), Ac-H3K9 (H9286, 1:1,000 dilution, Sigma-Aldrich), Drp1 (sc-271583, 1:1,000 dilution, Santa Cruz Biochemicals, Dallas, TX, USA), and Fis1 (sc-376447, 1:1,000 dilution, Santa Cruz Biochemicals). After a brief wash, membranes were incubated with horseradish peroxidase-conjugated IgG (Zymed, South San Francisco, CA, USA) for one hour at room temperature. Antibody signals were detected using an Las-4000 imager (GE Healthcare Life Science, Pittsburgh, PA, USA).

RNA isolation and real-time quantitative RT-PCR (qPCR)

Total RNA was extracted from skeletal muscle tissues using TRIzol reagent (Invitrogen). First-strand cDNA was generated using the random hexamer primer provided in the first-strand cDNA synthesis kit (Applied Biosystems, Foster City, CA, USA). Specific primers for each gene (Table S1) were designed using qPrimerDepot (http://mouseprimerdepot.nci.nih.gov). qPCR reactions were conducted in a final volume of 10 μl that contained 10 ng of reverse-transcribed total RNA, 200 nM of forward and reverse primers, and PCR master mix. qPCR was performed in 384-well plates using an ABI Prism 7900HT Sequence Detection System (Applied Biosystems). The mRNA level of each target gene of interest was normalized to that of *Gapdh* (in case of nuclear-encoded genes) or 16S rRNA (in case of mtDNA-encoded genes). For mitochondrial DNA content analysis, total DNA was extracted using a genomic DNA purification kit (Qiagen, Hiaden, Germany). Relative mtDNA was quantified by qPCR using primers for the mitochondrially encoded

gene cytochrome oxidase 2 (*Cox2*), normalized to the nuclear-encoded gene cyclophilin A (*Ppia*).

Indirect calorimetry

Mice were housed in an Oxymax/CLAMS metabolic cage system from Columbus Instruments (Columbus, OH, USA) with one mouse/chamber. Mice were placed in metabolic cages for one day to adapt and avoid stress during analysis. After 24-h acclimatization, mice were monitored continuously for 72 h with ad libitum feeding in an environmental room set at 20–23 °C with a 12 h–12 h (7:00 pm–7:00 am) dark-light cycle. The respiratory exchange ratio (VO_2/VCO_2) was measured using an Oxymax system. Data collected over the last 24 h of the experiment was used for analysis.

Mitochondrial respiration

C2C12 cells were obtained from ATCC (Manassas, VA, USA) and maintained in culture at <80% confluence in DMEM supplemented with 10% FBS. Differentiation of C2C12 cells was initiated by replacing 10% FBS by 2% horse serum (Gibco Life Technologies, Waltham, MA, USA). Differentiation media were changed every two days and cells at day 5 were considered as differentiated myotubes.

For Seahorse analysis (XF96, Agilent Technologies, Santa Clara, CA, USA), C2C12 myoblasts were seeded in XF24 plates. After six days of differentiation, C2C12 cells were treated overnight with vehicle (DMSO) or MDL801 (5 or 10 μM). One hour prior to beginning the assay, C2C12 myotubes were changed to DMEM containing with 5 mM glucose and 1 mM pyruvate. Oxygen consumption rate (OCR) was then measured according to manufacturer instructions with the injection of the Seahorse XF Cell Mito Stress Test Kit

(Agilent Technologies). Respiration was measured three times by injection of oligomycin (1 μM), fluoro-carbonyl cyanide phenylhydrazone (FCCP) (0.5 μM) and rotenone/antimycin A (1 μM). Data were normalized to protein content.

2. Supplementary Table

Table S1. Sequences and accession numbers for primers (forward, FOR; reverse, REV) used in real-time RT-PCR

Gene	Sequences for primers (5′ → 3′)
Myh7	FOR: ACAAGCTGCAGCTGAAGGTG
	REV: TCATTCAGGCCCTTGGCAC
Myh2	FOR: CCAGCTGCACCTTCTCGTTTGCCAG
	REV: CATGGGGAAGATCTGGTCTTCTT
Tnni1	FOR: TGAAGCCAAATGCCTCCACAACAC
	REV: ACACCTTGTGCTTAGAGCCCAGTA
Tnnc1	FOR: AGCTCATGAAGGACGGTGACAAGA
	REV: AACCGTGCAAGACCAGCATCTACT
Tnnt1	FOR: AAGGGGAGCGTGTGGATTTTG
	REV: TCCTCCTTTTTCCGCTGTTCA
Mb	FOR: CATGGTTGCACCGTGCTCACAG
	REV: GAGCCCATGGCTCAGCCCTG
Sdhb	FOR: CAGAGTCGGCCTGCAGTTTC
	REV: GGTCCCATCGGTAAATGGCA
Fndc5	FOR: TCCTCTTCATGTGGGCAGGT
	REV: GGGCTCGTTGTCCTTGATGATA
Nrf1	FOR: GGAGCACTTACTGGAGTCC
	REV: CTGTCCGATATCCTGGTGGT
Tfam	FOR: GCAAAGGATGATTCGGCTCAGGGAA
	REV: CCGGATCGTTTCACACTTCGACGG
Mtco1	FOR: CTACTATTCGGAGCCTGAGC
	REV: GCATGGGCAGTTACGATAAC
Mtco2	FOR: AACCATAGGGCACCAATGATAC
	REV: GGATGGCATCAGTTTTAAGTCC
Mcad	FOR: GGTTTGGCTTTTGGACAATG
	REV: TGACGTGTCCAATCTACCACA
Atp5o	FOR: TCTCGACAGGTTCGGAGCTT
	REV: AGAGTACAGGGCGGTTGCATA
Cox5b	FOR: TTCAAGGTTACTTCGCGGAGT
	REV: CGGGACTAGATTAGGGTCTTCC
Cycs	FOR: CCAAATCTCCACGGTCTGTTC
	REV: ATCAGGGTATCCTCTCCCCAG
Ndufs1	FOR: TGCAAATCCCTCGATTCTGTTAC
	REV: GCTTTCTCAATCTCTACCAGGC
Ndufv2	FOR: GCAAGGAATTTGCATAAGACAGC
	REV: TAGCCATCCATTCTGCCTTTG

Gene	Sequences for primers
Mfn1	FOR: TTGCCACAAGCTGTGTTCGG
	REV: TCTAGGGACCTGAAAGATGGGC
Mfn2	FOR: AGAGGCAGTTTGAGGAGTGC
	REV: ATGATGAGACGAACGGCCTC
Opa1	FOR: TCTGAGGCCCTTCTCTTGTT
	REV: TCTGACACCTTCCTGTAATGCT
Drp1	FOR: TCACCCGGAGACCTCTCATT
	REV: TGCTTCAACTCCATTTTCTTCTCC
Fis1	FOR: ACGAAGCTGCAAGGAATTTTGA
	REV: AACCAGGCACCAGGCATATT

3. Supplementary Figures

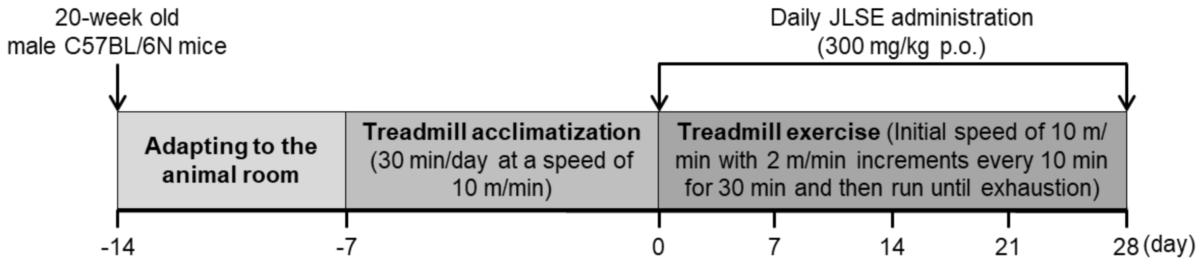

Figure S1. Schematic for exercise-to-exhaustion test.

Mice were acclimatized to the treadmill with a daily 30 min run at 10 m/min over 7 days. Mice were then subjected to a daily chronic treadmill running test over a 4 week period. The treadmill initially ran at 10 m/min, but this was increased by 2 m/min every 10 min until 16 m/min was reached, at which point the mice ran until exhaustion. At day 28, mice were subjected to indirect calorimetry study for 48 h (acclimatization to cage for 24 h and data collection during an additional 24 h). Muscle tissues were then harvested from mice after resuming exercise for 3-4 days following a calorimetry analysis. From ~ 5 tissues, parts was taken and immediately stored at -70 °C for protein/RNA extraction, and the remaining tissue parts were transferred to 30% sucrose solution and embedded with liquid nitrogen-cooled isopentane for histopathological analysis.

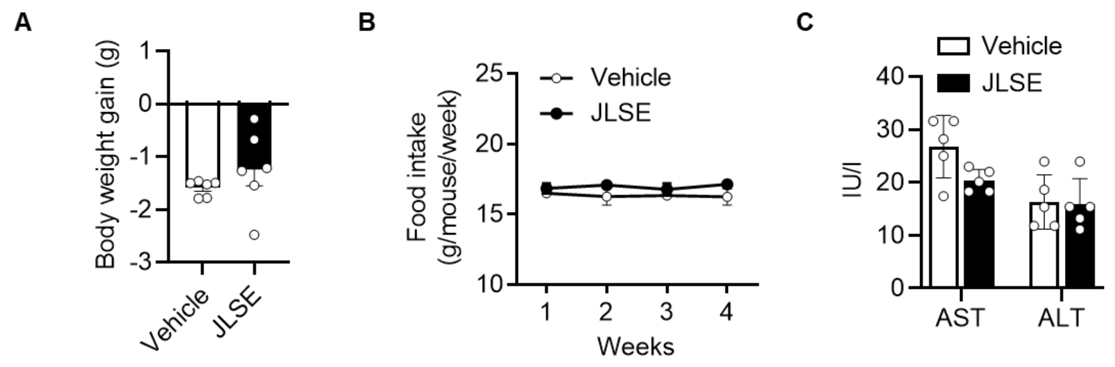

Figure S2. Effects of JLSE supplementation on body weight gain, food intake, and liver injury in exercise-trained mice. (**A, B**) Mice supplemented with PBS (vehicle) or JLSE (300 mg/kg) were placed on involuntary exercise training on a treadmill for 4 weeks. Body weights (n=5-6), food intake (n=5-6), and plasma levels of aspartate aminotransferase (AST) and alanine aminotransferase (ALT) (n=5) were determined. Values are mean±SD.

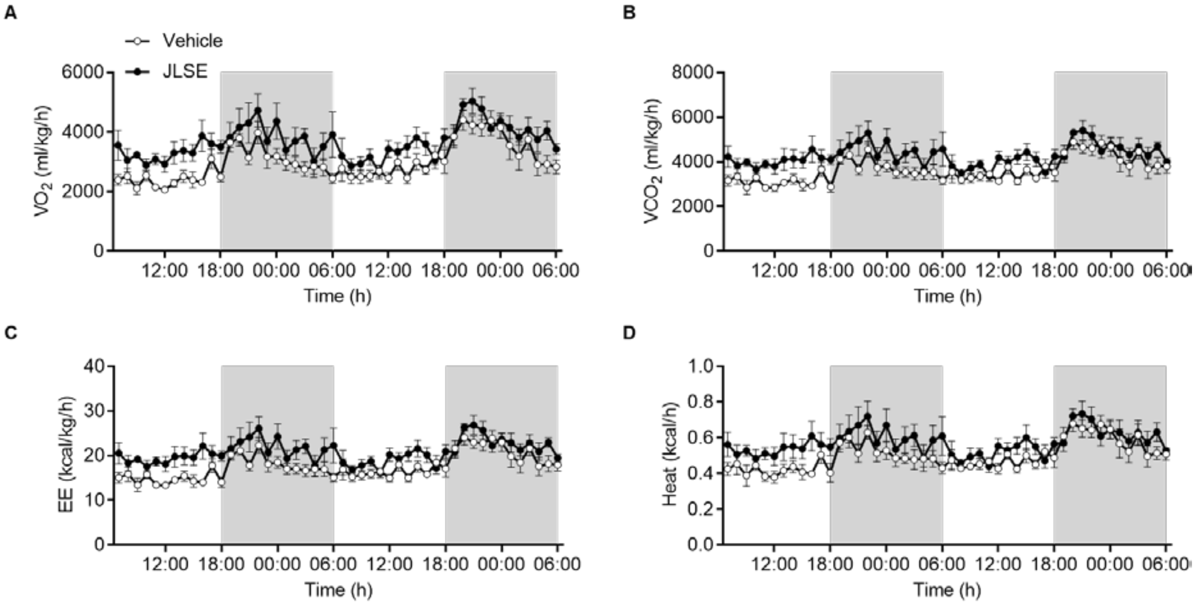

Figure S3. Indirect calorimetry analysis. All experimental procedures are identical to those described in Figure 3. Twenty-four hour VO_2 consumption rates (**A**), VCO_2 production rates (**B**), energy expenditure (**C**), and heat production (**D**) in mice (n=5). White and grey shaded areas represent light and dark, respectively. Values are mean±SD.

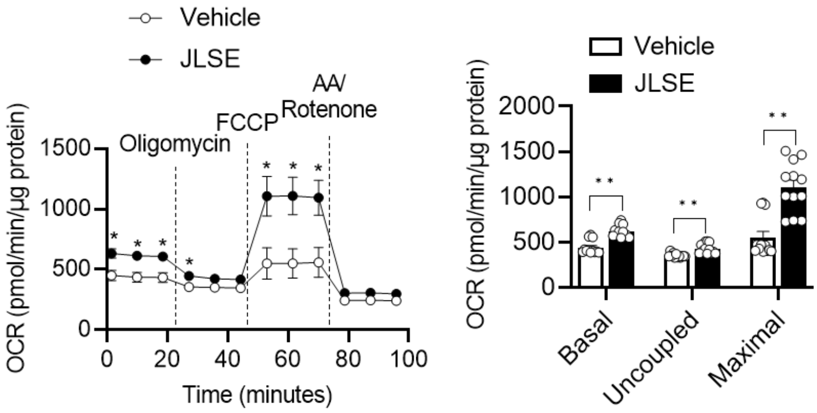

Figure S4. Measurement of oxygen consumption rate in C2C12 cells. Oxygen consumption rate (OCR) was measured using Seahorse XF analyzer in C2C12 cells. Basal respiration, respiration related to ATP production (uncoupled, difference between OCR before and after oligomycin injection), and maximal respiration (difference between OCR after FCCP and antimycin A (AA)/rotenone injection) were determined (n=12). Values are mean±SD.

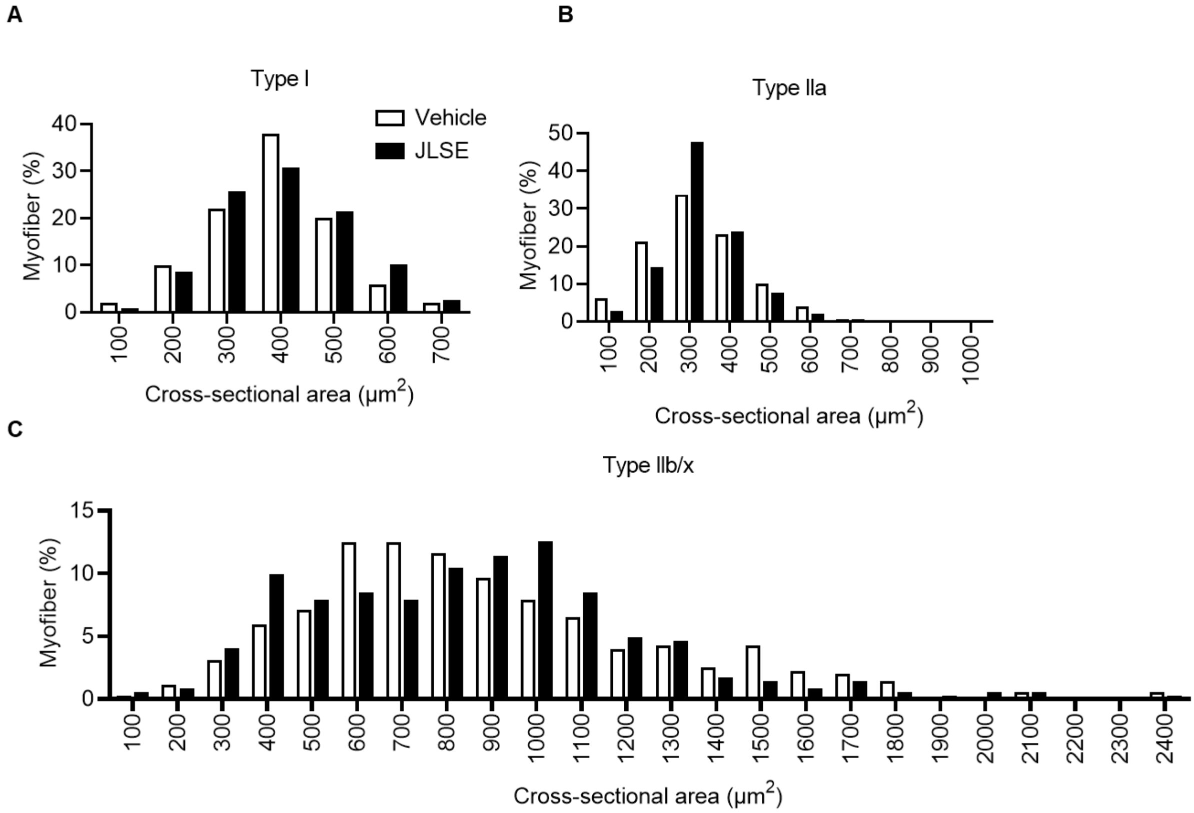

Figure S5. Histograms of myofiber size distributions of gastrocnemius muscles. On the basis of the expression of MyHC-positive myofibers (Figure 4B), the cross-sectional area of each type was determined.
